# Modeling and validating of oxygen transport in wave bioreactors: optimized experimental mass transfer method and novel Lattice-Boltzmann CFD approach

**DOI:** 10.3389/fbioe.2025.1688774

**Published:** 2026-01-21

**Authors:** S. Piontek, J. Fitschen, C. Weiland, T. Habicher, M. Schlüter, T. Wucherpfennig

**Affiliations:** 1 Boehringer Ingelheim Pharma GmbH & Co. KG, Biberach an der Riß, Germany; 2 Hamburg University of Technology, Hamburg, Germany

**Keywords:** wave bioreactor, CFD, volumetric mass transfer coefficient, rocked bioreactor, single use, process optimization

## Abstract

Wave bioreactors are commonly used in biopharmaceutical upstream processes as an intermediate stage between shake flasks and stirred tanks within the seed train. They offer a controlled environment for cell cultivation while minimizing shear stress. Accurate characterization of these systems is essential for optimizing cell culture performance, particularly as state of the art cell lines require higher volumetric mass transfer coefficients *k*_L_ *a*. This study aims to determine the volumetric mass transfer coefficient through experiments and computational fluid dynamics (CFD) simulations. An improved experimental method for the measurements of the volumetric mass transfer is presented, with results correlated to key process parameters: rocking angle, rocking rate, and filling volume. In addition, CFD simulations were caried out using M-Star CFD by means of a Lattice-Boltzmann Method-based solver. The mass transfer was calculated using Higbie’s penetration theory, incorporating the Kolmogorov scale to define contact time. The analysis also integrates concepts from Friedl and the surface renewal model, introducing the surface normal velocity as an additional parameter in the mass transfer coefficient *k*_L_ calculation. Analyzes were carried out for 10 and 50 L wave bioreactors, with one degree of freedom movement. Optimized process parameters were identified and validated in biological cultivations, resulting in increased dissolved oxygen levels in the medium. These findings contribute to improved characterization and control of wave bioreactors, enabling more accurate prediction of process parameter effects.

## 1 Introduction

Wave bioreactors were first introduced in the 1990s as a low-shear alternative to stirred tank bioreactors. These systems were specifically developed for the cultivation of highly shear-sensitive cells, including plant, insect, and animal cells. Previously, such cells could only be cultured in T-flasks or comparable setups, thereby restricting production volumes to one to two liters (Singh, 1999; Malla et al., 2021; Higbie, 1935; Maltby et al., 2016; Friedl, 2013; Kermani et al., 2011). The wave system comprises a single-use bag filled with medium and a rocking platform. The bag is placed on the temperature-controlled platform and can be agitated with varying tilt angles and frequencies, ensuring the necessary mixing and mass transfer. Aeration occurs through the headspace and therefore across the free surface (Eibl et al., 2009).

Currently, wave reactors are utilized in research applications for cell line and media development, as well as in biopharmaceutical production. They primarily serve as an intermediate stage within seed-train processes, bridging the gap between shake flasks and stirred tank reactors (Malla et al., 2021). Wave reactors can be operated in batch, fed-batch, and continuous modes, offering high flexibility depending on the desired application (Eibl et al., 2009). They are considered a type of single-use bioreactor, which has become increasingly utilized in recent years. These systems are delivered pre-cleaned and pre-sterilized, eliminating the need for separate cleaning procedures and thereby streamlining their integration into Good Manufacturing Practice (GMP)-compliant environments (Bartczak et al., 2023). The reactors are available in sizes ranging from V = 0.1 L (Bartczak et al., 2023) to V = 500 L (Seidel et al., 2022). The systems are engineered to function with either one or two degrees of freedom in their motion. Of these variants, models based on the original single-degree-of-freedom design (Eibl et al., 2009) remain predominant, while newer models with additional characteristics continue to be developed (Seidel et al., 2022).

Wave bioreactors have a high volume-to-surface ratio, but aeration relies solely on the headspace, so gas transfer to the liquid is limited by the surface area (Eibl and Eibl, 2007). Despite the movement allowing for a constant surface renewal, oxygen can be a limiting factor in cultivation in wave bioreactors with a one dimension of freedom movement (Seidel et al., 2022). This limitation can impact growth rates, posing a challenge for future projects that require higher oxygen demands due to increased productivity. Understanding the relevant process parameters and their specific effects can provide detailed insight into mass transfer and address potential limitations.

The volumetric mass transfer coefficient of wave bioreactors has historically been determined both experimentally and through CFD simulations. In experimental approaches, especially systems with one dimension of freedom have been characterized extensively (Singh, 1999; Mikola et al., 2007; Terrier et al., 2007; Eibl et al., 2009; Kalmbach et al., 2011; Junne et al., 2013; Marsh et al., 2017; Ghasemi et al., 2018; Pilarek et al., 2018; Bai et al., 2019b; 2019a; Zhan et al., 2019; Bartczak et al., 2023). For the determination of volumetric mass transfer the gassing-out method was employed in combination with the two-film theory by Lewis and Whitman. After stripping out oxygen from the liquid phase using nitrogen, the gassing was immediately switched to air sparging without prior degassing of the headspace. However, for the use of the two-film theory it is assumed that there is an ideally mixed gas phase (Emig et al., 2024). At the beginning of measurement with the described method, this assumption is not valid and varies with the gas flow rate. Such deviations have a notable impact on the calculated volumetric mass transfer coefficient. Therefore, there is a clear need for an enhanced experimental approach to accurately determine the volumetric mass transfer coefficient in wave bioreactors. Previous studies utilizing the aforementioned methodology have established correlations between the volumetric mass transfer coefficient and parameters such as rocking rate, rocking angle, and gas flow rate; however, filling volume has not been included as a correlation parameter (Pilarek et al., 2018). The aim of this experimental characterization of the volumetric mass transfer coefficient in the wave bioreactor system is to implement an improved experimental method overcoming the previous limitations described and establish a new correlation between process parameters based on the experimental results.

In addition to experimental characterization of wave bioreactors, a wide range of simulation-based data are available. These datasets were generated using various Navier-Stokes-based solvers, covering systems with both one (Öncül et al., 2010; Marsh et al., 2017; Zhan et al., 2019; Svay et al., 2020) and two degrees of freedom (Seidel et al., 2022). As an alternative to these traditional solvers, newer approaches based on the Lattice-Boltzmann-Method (LBM) are increasingly being used (Kuschel et al., 2021; Haringa, 2023; Kersebaum et al., 2024; Jamshidian et al., 2025). Operating at the mesoscopic scale, LBM enables accurate modeling of complex fluid systems (Krüger et al., 2017). Moreover, its localized and explicit structure allows for efficient parallelization, making it well-suited for GPU-based computation and significantly improving computational efficiency (Krüger et al., 2017). For this reason, the simulations presented in this study were conducted using a solver based on the Lattice-Boltzmann-Method. The results from the numerical simulation are in good agreement with the experimental results. Therefore, the LBM can be used for the simulation of wave bioreactors, reducing the computational effort in comparison to conventional RANS methods.

## 2 Materials and methods

This section is divided into two fundamental parts. The first part focuses on the experimental background. The second part outlines the CFD setup and explains the theoretical models that support the simulations.

### 2.1 Calculation of the volumetric mass transfer coefficient from experiments

For the experimental determination of the volumetric mass transfer, the gassing out method was implemented, using the two-film theory for the calculation. Afterwards the experimental data was correlated for the tested systems using the process parameters rocking rate, rocking angle and filling volume.

#### 2.1.1 Two-film theory

**FIGURE 1:**
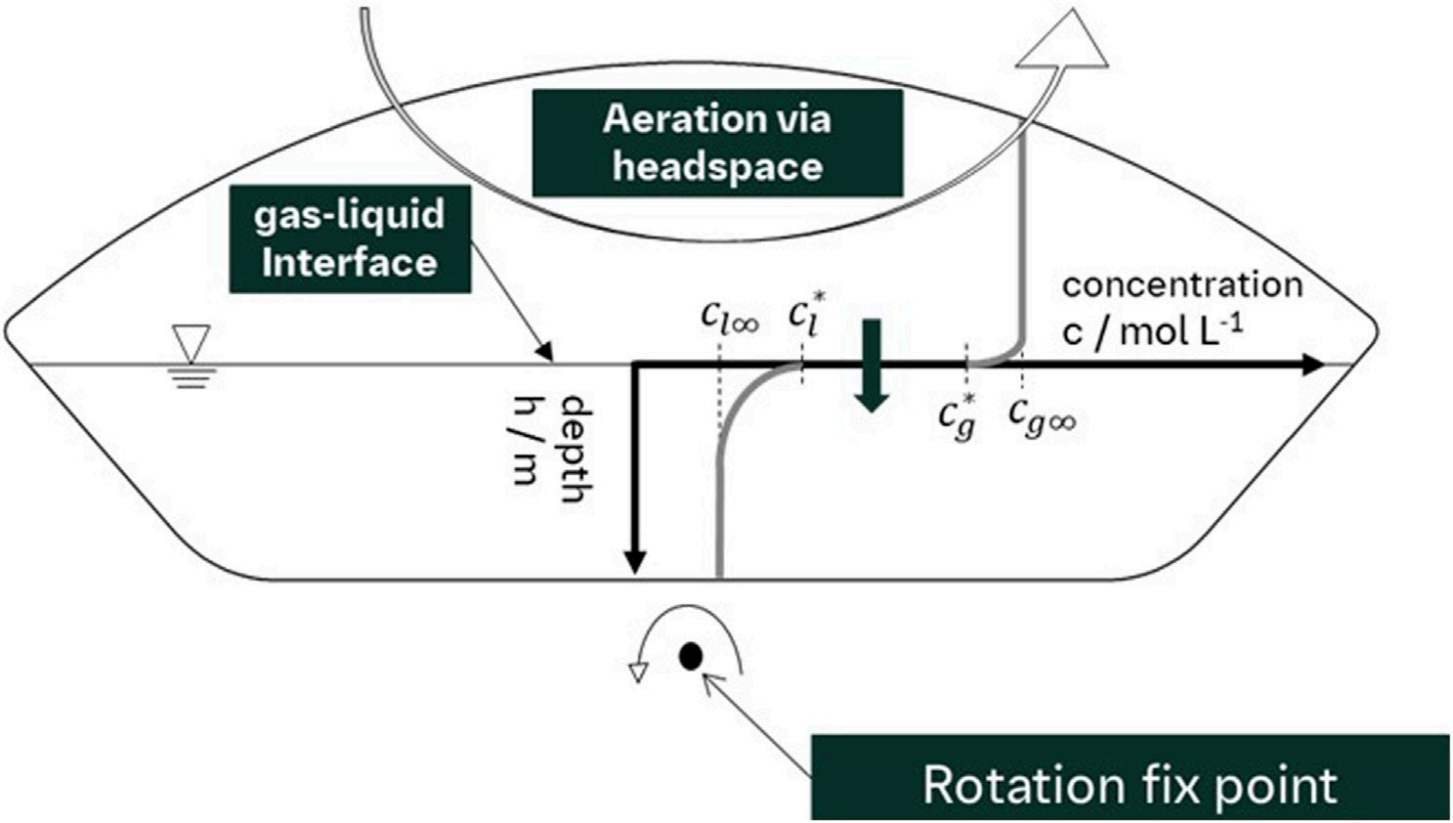
Schematic of gas–liquid mass transfer in a 50 L wave bioreactor during headspace aeration. The diagram illustrates the concentration gradient across the gas–liquid interface, with the labeled concentrations representing equilibrium and bulk values in both phases. The rotation fix point is shown at the base, reflecting the rocking motion characteristic of wave bioreactors.

The Lewis–Whitman two-film theory builds on the original film theory and is widely used to describe mass transfer between two phases. It assumes that transfer happens linearly across two thin boundary layers—one in each phase—at the interface at steady state. While the resistance to mass transfer is mainly on the liquid side, the actual movement occurs from the gas phase into the bulk liquid. To implement the model, it is assumed that the interface does not impose resistance to mass transfer and that the boundary layers exhibit laminar flow characteristics, where molecular diffusion is the primary transport mechanism (Emig et al., 2024). In the wave reactor, it is additionally assumed that the gas phase in the headspace is an ideal mixed gas, resulting in a constant oxygen concentration in the air as shown in Figure 1.

Furthermore, the mass transfer from the liquid to the gas phase is assumed as negligible. Under these assumptions, the volumetric mass transfer coefficient can be determined by the temporal increase in the dissolved gas concentration in the liquid phase in relation to the maximum gas saturation concentration, as shown in Equation 1.

$$k_{L}a=-\ln\left(\frac{c^{*}-c\left(t\right)}{c^{*}-c_{0}}\right)\cdot \;\frac{1}{t-t_{0}}$$ (1)

#### 2.1.2 Correlation of volumetric mass transfer and experimental data using process parameters

In 1983, Van’t Riet showed that the volumetric mass transfer coefficient is dependent on various operational parameters in an aerated stirred tank reactor (Riet, 1983). Riet identified the dependence of the volumetric mass transfer coefficient on power input, aeration rate, and fill volume, as shown in Equation 2.

$$k_{L}a=C_{1}\cdot {\left(\frac{P}{V}\right)}^{\alpha 1}\cdot q^{\beta 1}\cdot V^{\gamma }$$ (2)

This finding is now widely recognized and serves to characterize aerated stirred tank reactors. Over time, similar correlations have been developed for other systems, each referencing relevant operational parameters. In large-scale cylindrical vessels with a flat-bottom surface the vessel diameter *d*_*V*_, shaking frequency *n*, fill volume *V*, dynamic viscosity *ν*, diffusion coefficient *D*_*L*_, and gravity *g* have been identified as influencing parameters, as shown in Equation 3 (Klöckner et al., 2013).

$$k_{L}a=1.06\cdot d_{V}^{4.3}\cdot n^{2.12}\cdot V^{-1.2}\cdot \;\nu^{-0.21}\cdot D_{L}^{0.12}\cdot \;g^{-0.51}$$ (3)

A similar structure can be observed for shake flasks, with the addition of shaker diameter, as shown in Equation 4 (Henzler and Schedel, 1991; Dinter et al., 2025). Correlations even reach the smallest scale, with comparable equations for microtiter plates (Doig et al., 2005).

$$k_{L}a=0.5\cdot \;{d_{s}}^{\left(\frac{73}{36}\right)}\cdot \;n\cdot \;V^{\left(-\frac{8}{9}\right)}\cdot \;\nu_{L}^{\left(-\frac{13}{54}\right)}\cdot \;D_{L}^{\left(\frac{1}{2}\right)}\cdot \;g^{\left(-\frac{7}{54}\right)}$$ (4)

For wave bioreactor systems, a correlation has been established considering the rocking rate, rocking angle and surface aeration as shown in Equation 5 (Pilarek et al., 2018).

$$k_{L}a=10.379\cdot \;\omega^{0.594}\cdot \;{\left(\sin\;\alpha \right)}^{0.443}\cdot \;q^{0.592}$$ (5)

#### 2.1.3 Experimental set-up

The experimental determination of the volumetric mass transfer coefficient started with the selection of a reference medium to assess comparability with the actual cultivation medium. Subsequently, mass transfer was quantified in wave bioreactors under varying process conditions, followed by the implementation of enhanced parameters for CHO cell cultivation.

The comparability of different media was previously evaluated, not in a wave bioreactor, but in a sparged 2 L stirred tank reactor at various agitation and gas flow rates, though these results have not been published. Here, a buffer system was selected to closely replicate Boehringer Ingelheim cultivation medium for CHO-K1 cell lines. PBS plus Pluronic (AppliChem) served as the tested reference medium and was compared to the actual culture medium, with detailed compositions provided in the Supplementary Material. The use of any additive, including PBS, increases mass transfer resistance *β*_L_ and reduces the mass transfer coefficient *k*_L_, in wave bioreactors, this leads to a decrease in the overall volumetric mass transfer coefficient *k*_L_ *a* (Wierzchowski et al., 2021). The addition of Pluronic further impairs mass transfer (Elibol, 1999; Sieblist et al., 2013). The use of PBS and Pluronic as the test medium aimed to make the study more industrially relevant than using only water.

After selecting the test medium, volumetric mass transfer coefficients were measured in both 10 L and 50 L wave bioreactors. The experiments followed a full factorial design, using three levels for each factor: filling volumes of 15, 20, and 25 L, rocking rates of 17, 24, and 31 rpm, and rocking angles of 4°, 7°, and 10°. These combinations were tested in both bioreactor sizes. In the 50 L bioreactor, additional intermediate points were included to improve model coverage and accuracy. Specifically, rocking rates of 27 and 29 rpm and a rocking angle of 9° were investigated only in the larger vessel. Replicates were performed at central conditions such as 24 rpm, 7°, and 20 L to assess variability and strengthen statistical confidence. In total, 42 experimental runs were completed with the 50 L wave and 34 runs were conducted with the 10 L wave. In order to minimize bias, a partially randomized order of experiments was conducted, by grouping the experiments by volume, but randomizing the order of tested rocking rates and angles. In addition, a switch between working volumes was conducted switching from 20 L to 15 L–25 L and back to 15 L while conducting the experiments. This approach enabled the evaluation of main effects and interactions between factors, supporting robust regression analysis. The resulting model is valid within the tested ranges for each parameter, and predictions outside these ranges should be interpreted with caution.

During the measurements a gas flow rate of 20 L/h was used for all experiments. In order to understand the effect of the gas flow rate on the volumetric mass transfer coefficient, tests were conducted with a gas flow rate of 120 L/h and 140 L/h. Parameter combinations of rocking rate = 31 rpm and rocking angle = 7° at 140 L/h gas flow rate and rocking rate = 31 rpm and rocking angle = 10° at 120 L/h gas flow rate were selected. The experiments only focused on additional gas flow rates grater *q* = 20 L/h because in cultivation lower gas flow rates are not feasible. This analysis was done to determine if the gas flow rate would be a fourth fitting parameter to be considered within the design of experiment.

For the experimental determination of the volumetric mass transfer coefficient the system was then stripped with maximum nitrogen until the dissolved oxygen (DO) level dropped below 10%. Subsequently, the headspace was evacuated using a vacuum pump and inflated with maximum air at a flow rate of 120 L/h. The exhaust valve was opened once the pressure in the headspace exceeded 10 mbar. Measurements were initiated as a new experiment, with rocking and angle adjustments made according to DOE specifications. The experiment was terminated when the DO level exceeded 80% or the time surpassed 40 min. Upon conclusion of the measurement, the process was repeated from the initial step for subsequent experiments. The DO concentration was measured using dissolved oxygen sensitive spots SP-PSt3-NAU by PreSens (sensor response time is less than 6 s), placed directly inside the bag, delivering an inline signal. Based on the data, the volumetric mass transfer coefficient was calculated using Equation 1.

The determined volumetric mass transfer coefficients were used for a correlation of chosen process parameters as further explained in the results part. The correlation data was then used to determine process parameters aiming at an enhancement of the volumetric mass transfer coefficient of 50% and 75% in the 50 L wave bioreactor. In order to validate the results, a proprietary CHO-K1 glutamine synthetase (GS)−/− cell line producing a recombinant protein was cultivated over a 3-day cultivation time using a rocking rate of 24 rpm, filling volume of 20 L and a rocking angle of 7°. As enhanced process conditions an increase of the rocking angle was chosen and changed from 7° to 9°. As a second improvement an increase in rocking angle from 7° to 9° as well as an increase in rocking rate from 24 rpm to 26 rpm was chosen. In order to compare the results, a *pO*_*2*_ inline measurement was conducted over the entire cultivation time. As a media a Boehringer Ingelheim owned CHO cell media with a proprietary formulation was used. The pH range for this unit operation is defined as 7.10–6.95 (+/- 0.25).

#### 2.1.4 Tested systems

**FIGURE 2:**
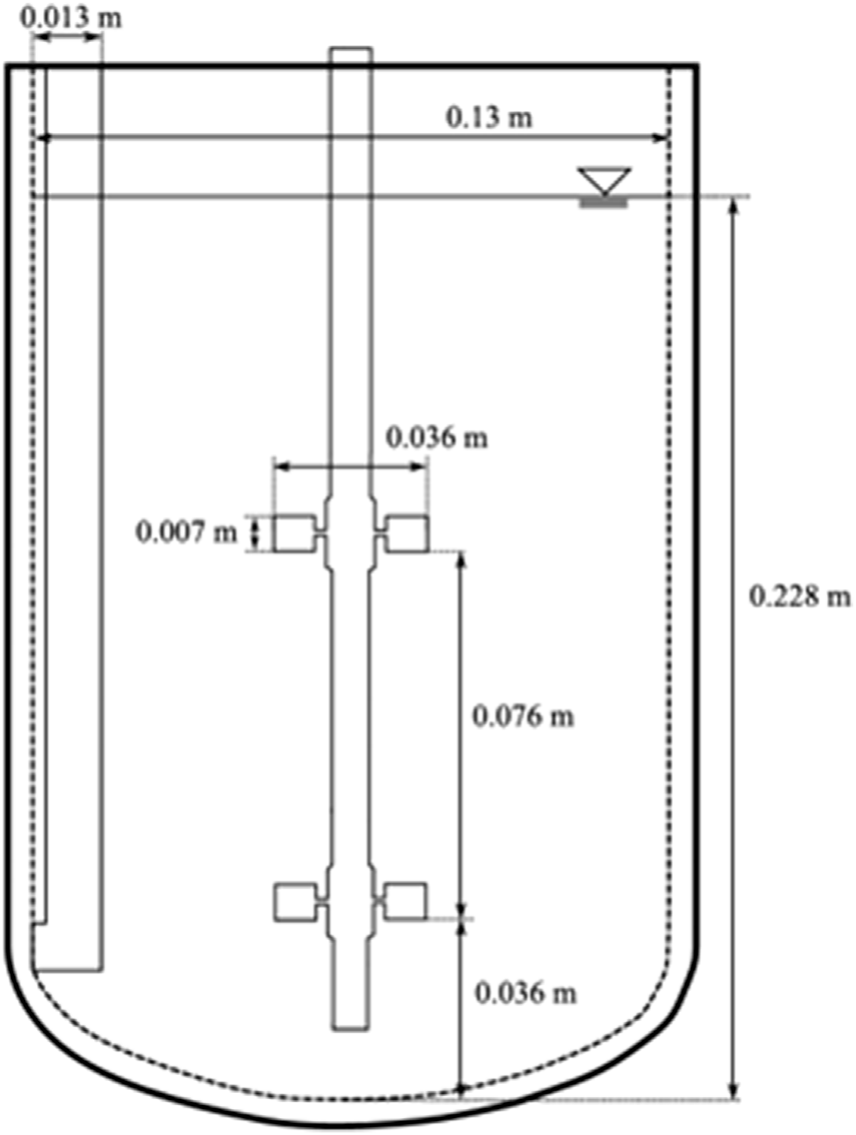
Geometry and dimensions of a 2 L stirred bioreactor as previously published in (Weiland et al., 2023).

**FIGURE 3:**
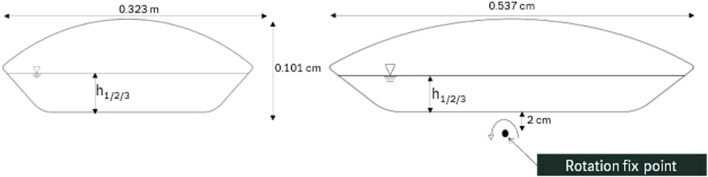
Geometry and dimensions of the 10 L wave reactor with designated rotation axis. The left panel presents the front view of the wave reactor, while the right panel displays the side view with rotation fix point.

**FIGURE 4:**
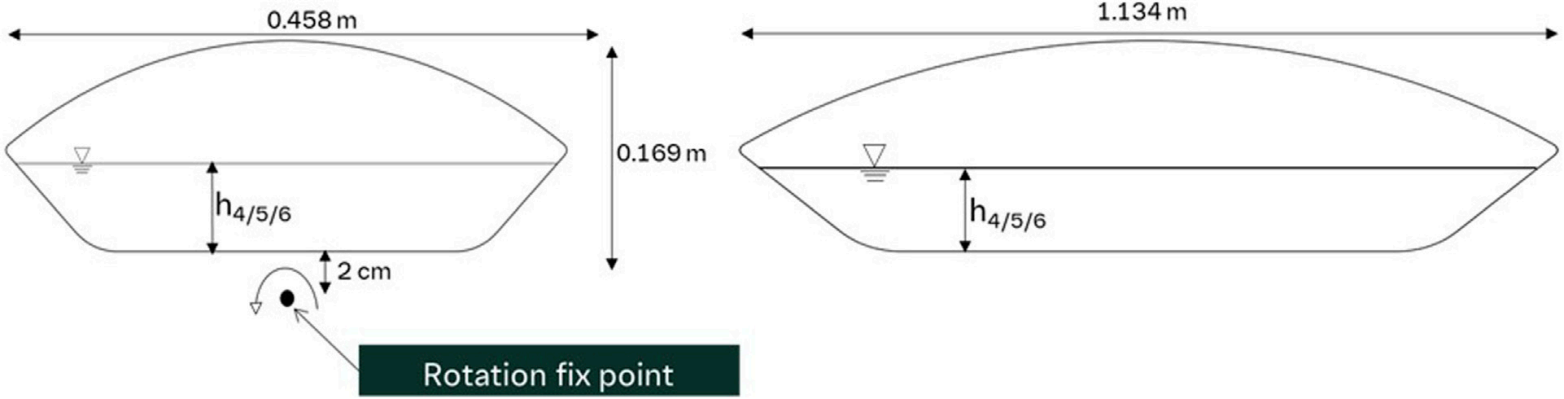
Geometry and dimensions of the 50 L wave reactor with rotation fix point. The left panel presents the front view of the wave reactor with rotation fix point, while the right panel displays the side view.

The three systems tested for all experiments are shown in Figures 2–4 starting with the depiction of the 2 L stirred bioreactor in Figure 2, used for the determination of the volumetric mass transfer in PBS and Pluronic in comparison to the volumetric mass transfer in Boehringer Ingelheim cultivation medium.

The primary emphasis of this study lies on the wave bioreactor systems. Here, the Sartorius Biostat® RM platform was used alongside a 10 L and a 50 L wave bioreactor. The configuration and dimensions of the 10 L unit are shown in Figure 3. Notably, the rotational axis of the 10 L bioreactor is positioned along the longer side of the bag.

In Figure 4, the configuration and dimensions of the 50 L unit are displayed. Here, the rotational axis is positioned along the short side of the bag.

Table 1 lists the different filling heights for both the 10 L and 50 L wave bioreactors at different filling volumes tested.

**TABLE 1:** Filling height of liquid for the 10 L and 50 L bioreactor at different filling volumes.

Symbol	Bioreactor size/L	Filling volume/L	Filling height/m
h_1_	10	3	0.0266
h_2_	10	4	0.0343
h_3_	10	5	0.0411
h_4_	50	15	0.0457
h_5_	50	20	0.0580
h_6_	50	25	0.0710

### 2.2 Calculation of the volumetric mass transfer from CFD

For the CFD simulations the commercially available software M-Star CFD (M-Star Computational Fluid Dynamics (CFD) Software, 2025) was used. The focus was the 50 L wave bioreactor, because of the greater need for enhanced process parameters. The 10 L wave bioreactor was tested for a chosen number of process parameters. The generation of the geometric model, as well as the simulation setup is described in the following subsection.

#### 2.2.1 Wave bioreactor model

For the generation of the CAD models, the 10 and 50 L wave bioreactor were each subjected to 3D scanning. Despite the inherent flexibility of the bags, experimental observations indicated that the geometric changes of the bags, due to the constant operational overpressure, can be considered negligible (Öncül et al., 2010).

For the 50 L system simulations were carried out for all process parameters and filling volumes as described in Section 2.1.1. The 10 L system was tested for frequencies at all ranges, including lowest and highest and rocking angle and rate combinations, as well as the middle point. Those parameter combinations being 24 rpm and 7°, 17 rpm and 4° and 31 rpm and 10° for all filling volumes.

#### 2.2.2 Simulation

The software M-Star CFD version 3.12.76 was used in order to perform the simulations for all cases tested. M-Star thereby numerically solves the Lattice Boltzmann equation, shown in Equation 6 (Krüger et al., 2017). The Lattice Boltzmann Method is characterized by its strictly local formulation in both space and time, which allows it to inherently preserve mass and momentum with a level of precision constrained only by the limits of numerical resolution. The fundamental equation governing this method captures the evolution of particle distribution functions through discrete streaming and collision steps.

$$f_{i}\left(x+c_{i}\Delta t,t+\Delta t\right)=f_{i}\left(x,t\right)+\Omega_{i}\left(x,t\right)$$ (6)

In this equation $f_{i}\left(x,t\right)$ represents the distribution function of the particles at position *x* and time *t*, while $f_{i}\left(x+c_{i}\Delta t,t+\Delta t\right)$ denotes its value after a time step Δt. The term $\Omega_{i}\left(x,t\right)$ refers to the collision operator, which is responsible for alterations resulting from particle interactions. For this, both the space and time resolution must be specified (Thomas et al., 2021).

For the numerical set-up a resolution of *L*_x_= 400 was chosen, leading to a lattice spacing of dx= 0.0011 m, based on a grid study performed between *L*_x_ = 100 and *L*_x_ = 700. The study showed no change in the fluid velocity starting at a resolution of *L*_x_ = 400, as provided in the Supplementary Material. The temporal resolution Δ*t* was specified depending on the maximum velocity of the bag movement, dependent on the rocking rate *ω*, rocking angle *α*, Courant Number *Co* and rocking radius *r* between the motion axis and the farthest point of the wave geometry on the same axis and the length of the wave bioreactor *L*, calculated by Equations 7–10.

$$\frac{dv}{dt}=\max_{t}\left[\pi \left(\frac{\alpha \cdot \pi }{180}\right)\cdot \left(2\pi \cdot \omega \right)\cdot \cos\left(2\pi \cdot \omega \cdot t\right)\right]$$ (7)

Here, Equation 7 calculates the maximum acceleration of the bag movement *v* over time, incorporating the rocking angle *α*, rocking rate *ω*, and time-dependent cosine modulation. From that, the time dependent movement in *z* direction d*z*/d*t* was calculated using Equation 8.

$$\frac{dz}{dt}=2\pi \cdot \frac{da}{dt}+r$$ (8)

It combines the angular contribution of the rocking motion with the geometric influence of the rocking radius *r*. In addition, the resolution dependent change in *x* was calculated using Equation 9.

$$dx=\frac{L}{L_{x}}$$ (9)

The spatial step size dx was determined by dividing the total length of the wave bag in the x-direction by the chosen grid resolution L_x_. Results from Equations 8, 9 were then used to determine the temporal resolution needed coupled to the wave bioreactor movement, to ensure comparability between the tested setups, based on the maximum movement.

$$dt=\text{Co}\cdot \frac{dx}{dz\cdot dt^{-1}}$$ (10)

Here, the Courant number was multiplied with the ratio of d*x* and d*z* to the time increment d*t*. For both systems a Courant number of *Co* = 0.05 was chosen for the calculation. Both systems showed a motion radius *r* = 0.2425 m, because the 50 L system is rocked around the x-axis, the 10 L system is rocked around the y-axis. The length of the x-axis differs, with *L*_50L_ = 0.46 m for the 50 L wave system and *L*_10L_ = 0.32 m for the 10 L wave system.

For the model, the free surface model was chosen using the Large Eddy simulation to resolve the turbulence at the interface between fluid and gas phase.

#### 2.2.3 Calculation of the volumetric mass transfer coefficient from CFD

The volumetric mass transfer coefficient can be calculated by multiplying the mass transfer coefficient *k*_L_ and the specific surface area *a*.

To calculate mass transfer, several approaches were considered. These include elements of Higbie’s penetration model, with the Kolmogorov scale for the time resolution, as well as additional factors proposed by Friedl (Friedl, 2013). Higbie’s model, originally developed to describe mass transfer at gas bubbles, is presented in Equation 11. In this context, the temporal resolution is based on the Kolmogorov length scale (Higbie, 1935; Maltby et al., 2016).

$$k_{L}=\frac{2}{\sqrt{\pi }}\cdot \sqrt{D_{L}\sqrt{\frac{\varepsilon }{\nu }}}$$ (11)

In this context, *D*_*L*_ denotes the diffusion coefficient, ε represents the eddy dissipation rate, and ν is the kinematic viscosity of the liquid phase. Since this model primarily targets air bubbles, additional parameters relevant to the mass transfer in the wave bioreactor were incorporated. Friedl demonstrated that the rate of mass transfer correlates with the interfacial contact velocity between the fluid and gas phases, described by the friction velocity. Friedl’s investigations focused on mass transfer at wave surfaces within a wind tunnel environment (Friedl, 2013).

Given that velocity serves as a useful proxy for estimating contact time at the interface, a comparable parameter was identified for the wave bioreactor. The normal velocity component perpendicular to the surface was defined as the most suitable measure. This parameter characterizes vertical advection at the interface, which is particularly relevant during the initial phase of surface renewal (Kermani et al., 2011). An increased normal velocity at the surface reduces the surface age and enhances the renewal rate, thereby positively influencing the mass transfer coefficient. The normal velocity was determined by calculating the magnitude of the product of the velocity vector and the surface normal for every surface point separately, as shown in Equation 12.

$$v_{n}=\left|v\cdot n\right|$$ (12)

This parameter was subsequently incorporated into the calculation of the mass transfer coefficient *k*_L_, with the addition of a system-specific fitting parameter *c* varying for the systems with c_50_ = 72.61 and c_10_ = 859.47, as shown in Equation 13.

$$k_{L}=\frac{v_{n}}{c}\cdot \sqrt{D_{L}\sqrt{\frac{\varepsilon }{\nu }}}$$ (13)

**FIGURE 5:**
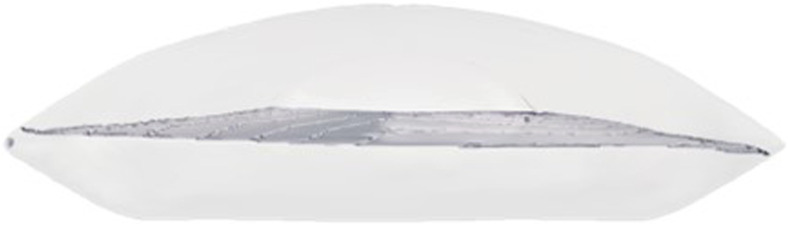
Model of a 50 L wave bioreactor with a 20 L working volume, visualized in ParaView. The interface between the liquid and gas phases was isolated using the Volume of Fluid (VOF) method, with a volume fraction range of 0.45–0.95.

The interface was identified using the Volume of Fluid (VOF) method (Tryggvason et al., 2009; Soh et al., 2016) and prefiltered within a volume fraction range of 0.45 ≤ α_L_ ≤ 0.95 to ensure a single-layer lattice structure at the surface as shown in Figure 5, determined empirically.

To approximate the interfacial area *a* of lattice cell *i*, the magnitude of the gradient of the volume fraction within the cell was used, as described in Equation 14, published and described by Soh as well as used by Wutz and Dinter (Soh et al., 2016; Wutz et al., 2018; Dinter et al., 2025).

$$a_{i}=\left|\nabla a_{i}\right|$$ (14)

The calculation of the volumetric mass transfer coefficients was performed across the entire interface on a per-cell basis, as described in Equation 15.

$$k_{L}a=\frac{\int_{V}k_{L,i}\;\cdot a_{i}\cdot dV}{V}$$ (15)

## 3 Results

The following section contains experimental and numerical results as well as the implementation of improved process parameters.

### 3.1 Experimental results

**FIGURE 6:**
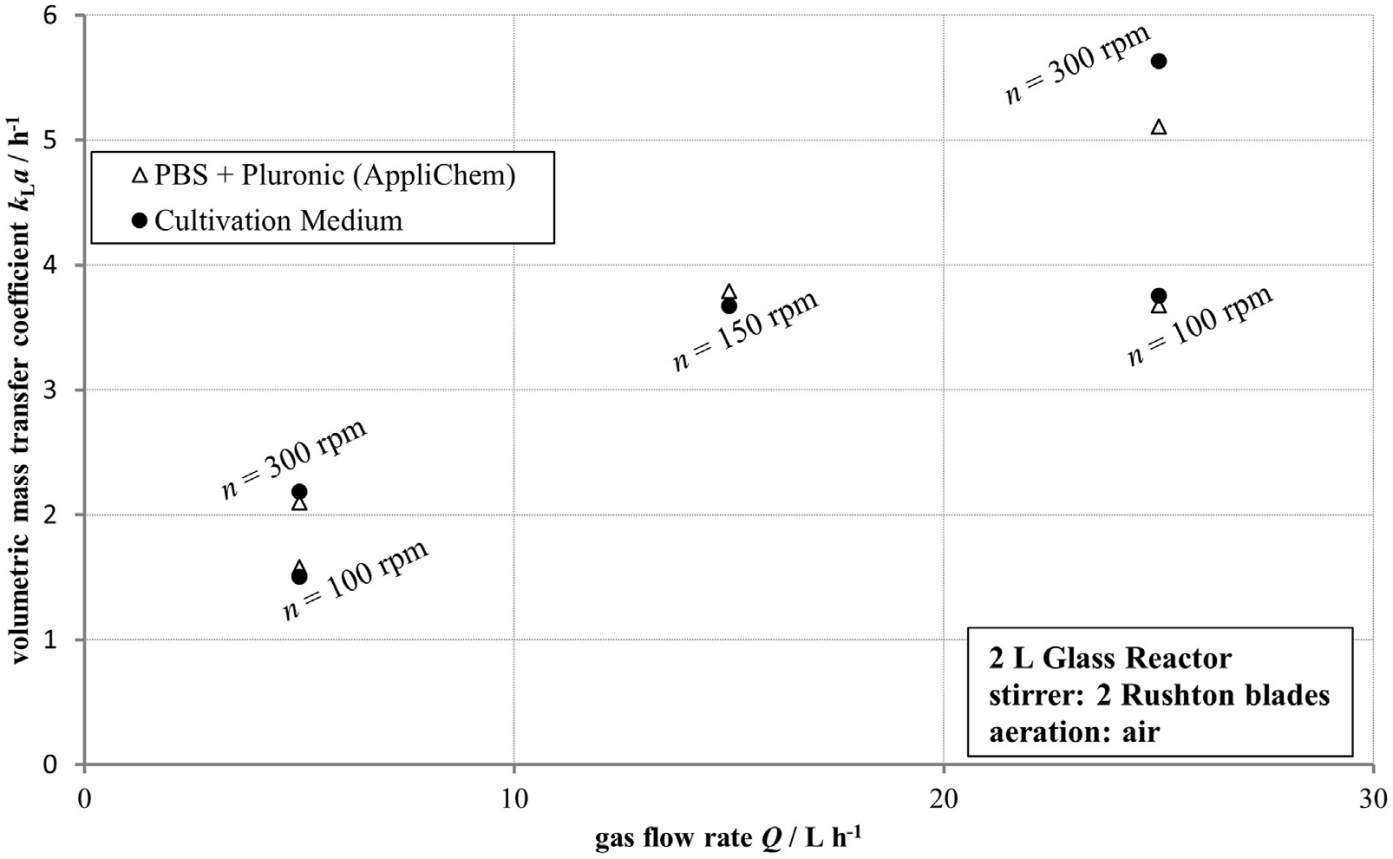
Volumetric mass transfer coefficient *k*_*L*_ *a,* determined in a 2 L glass reactor using a PBS + Pluronic medium by AppliChem, as well as a proprietary cultivation medium by Boehringer Ingelheim at varying gas flow rates and stirrer speeds, plotted against the gas flow rate.

Comparable volumetric mass transfer coefficients were determined in a 2 L stirred glass reactor for both PBS supplemented with Pluronic, and the proprietary cultivation medium developed by Boehringer Ingelheim across various aeration rates and impeller speeds. Only at the highest aeration rate and agitation speed were minor differences detected. Overall, PBS + Pluronic was confirmed to be a representative medium with respect to volumetric mass transfer behavior, as can be seen in Figure 6.

The results of the improved experimental determination of the volumetric mass transfer coefficient, achieved by degassing the headspace following outgassing, as well as the outcomes of the cultivation experiments, are presented in the following section.

**FIGURE 7:**
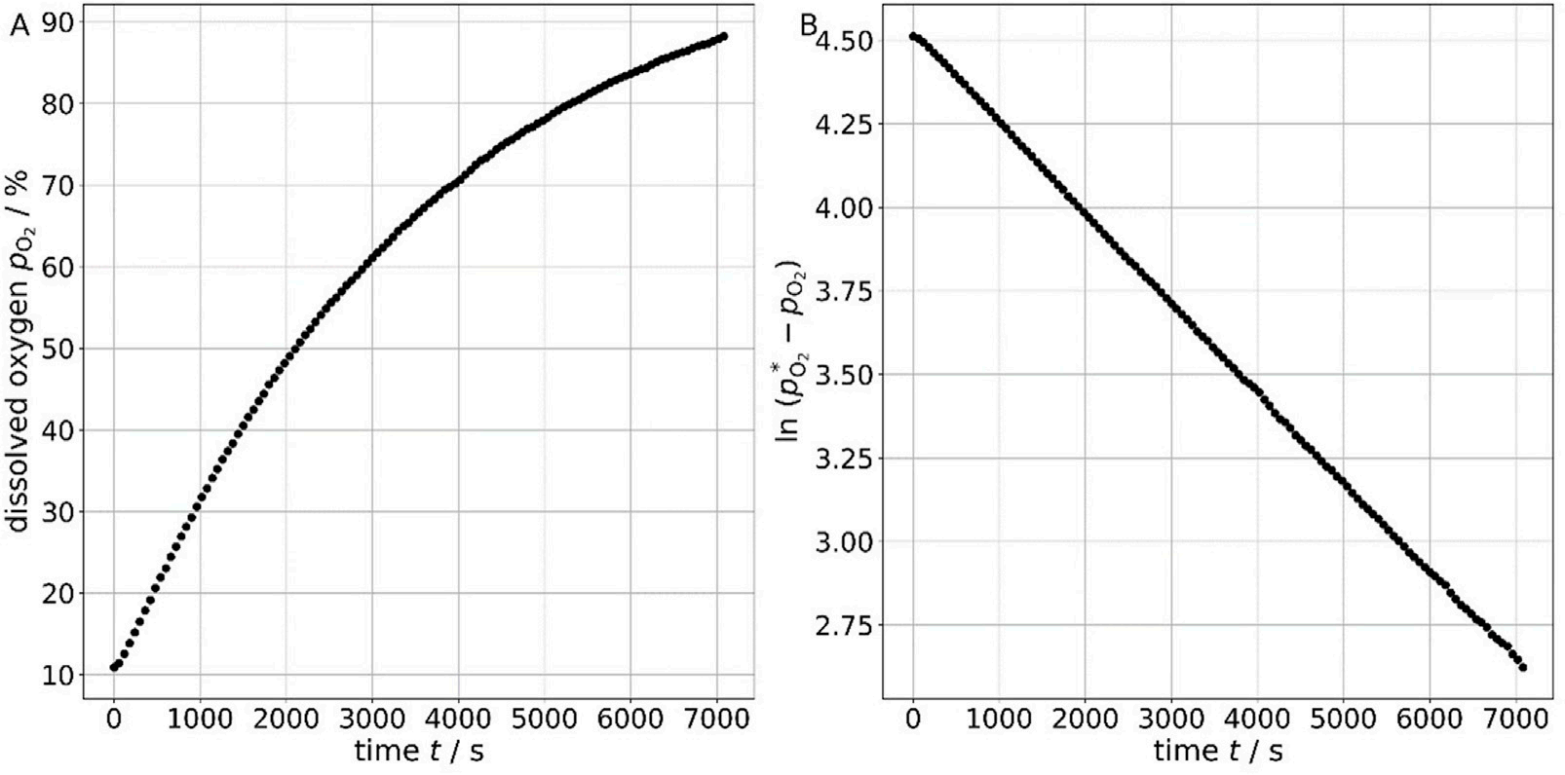
Increase in dissolved oxygen concentration over time **(A)**, and the natural logarithm of the difference between the maximum dissolved oxygen concentration and the measured values over time, where the slope corresponds to the negative value of the volumetric mass transfer coefficient **(B)**.

The volumetric mass transfer coefficient was determined based on the increase in dissolved oxygen concentration in the liquid phase over time, as illustrated in Figure 7. The slope of the natural logarithm of the difference between the maximum dissolved oxygen *pO*_*2*_ * and the measured dissolved oxygen *pO*_*2*_ over time corresponds to the negative value of the volumetric mass transfer coefficient *k*_L_ *a*.

Several factors were considered in evaluating the correlation of process parameters. A previously published correlation included not only the rocking angle and rocking rate but also the aeration rate (Pilarek et al., 2018). However, this observation could not be confirmed using the given experimental method. Experiments conducted at constant rocking angle and frequence, but with aeration rates of 20 L/min and 120 L/min, showed no significant change in the volumetric mass transfer coefficient.

**FIGURE 8:**
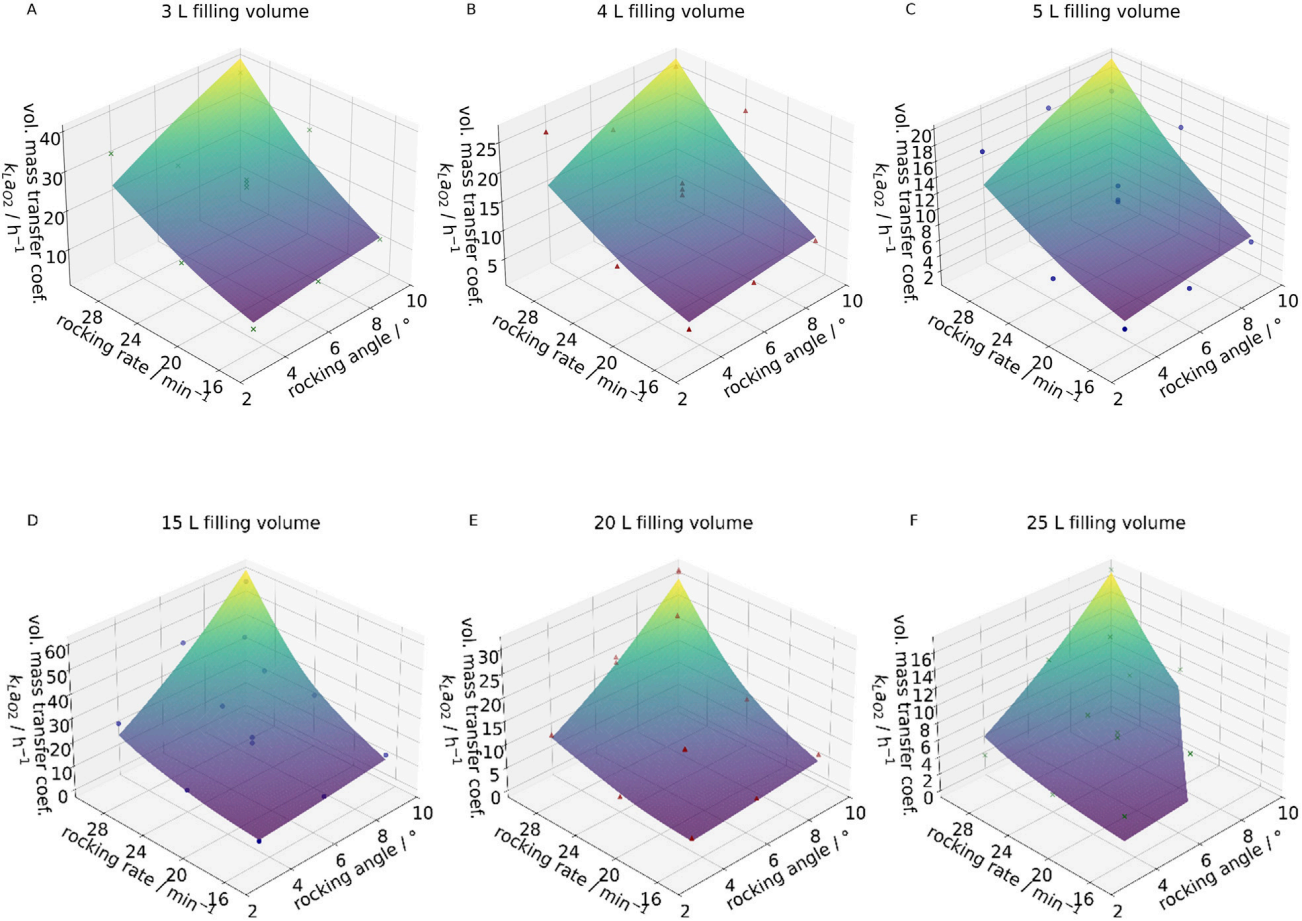
Response surface plots of the correlated data (data points) and the experimental data (surface) for the 10 L wave bioreactors at 3 L filling volume **(A)**, 4 L filling volume **(B)** and 5 L filling volume **(C)**, as well as the 50 L wave bioreactor at 15 L filling volume **(D)**, 20 L filling volume **(E)** and 25 L filling volume **(F)**.

In contrast, the fill volume exhibited a substantial influence, as it strongly affects the specific surface area. Consequently, it was introduced as an additional system-specific fitting parameter. The response surface plots for different fill volumes in the 10 L and 50 L wave bioreactors are shown in Figures 8A–F, respectively.

For the fitting, the parameters rocking frequence, rocking angle and filling volume were determined as relevant fitting parameters for the systems. The fitting was performed individually for each system. For the 10 L wave bioreactor, this resulted in a correlation of the form applicable for rocking rates between 17 and 31 rpm, rocking angles between 4° and 10° and filling volumes between 3 L and 5 L:

$$k_{L}a=9.77\cdot 10^{-3}\cdot \;\omega^{2.41}\cdot \alpha^{0.67}\cdot V^{-1.39}$$

For the 50 L wave bioreactor, the resulting correlation equation took the form applicable for rocking rates between 17 and 31 rpm, rocking angles between 4° and 10° and filling volumes between 15 L and 25 L:

$$k_{L}a=1.2\cdot 10^{-3}\cdot \;\omega^{3.89}\cdot \;\alpha^{1.55}\cdot \;V^{-2.26}$$

A significantly stronger influence of all operating parameters on the volumetric mass transfer coefficient was observed in the 50 L system compared to the 10 L system. The expected trends were confirmed: increases in rocking rate and angle had a positive effect, while an increase in fill volume negatively impacted the volumetric mass transfer coefficient. The latter is attributed to its effect on the specific surface area.

**FIGURE 9:**
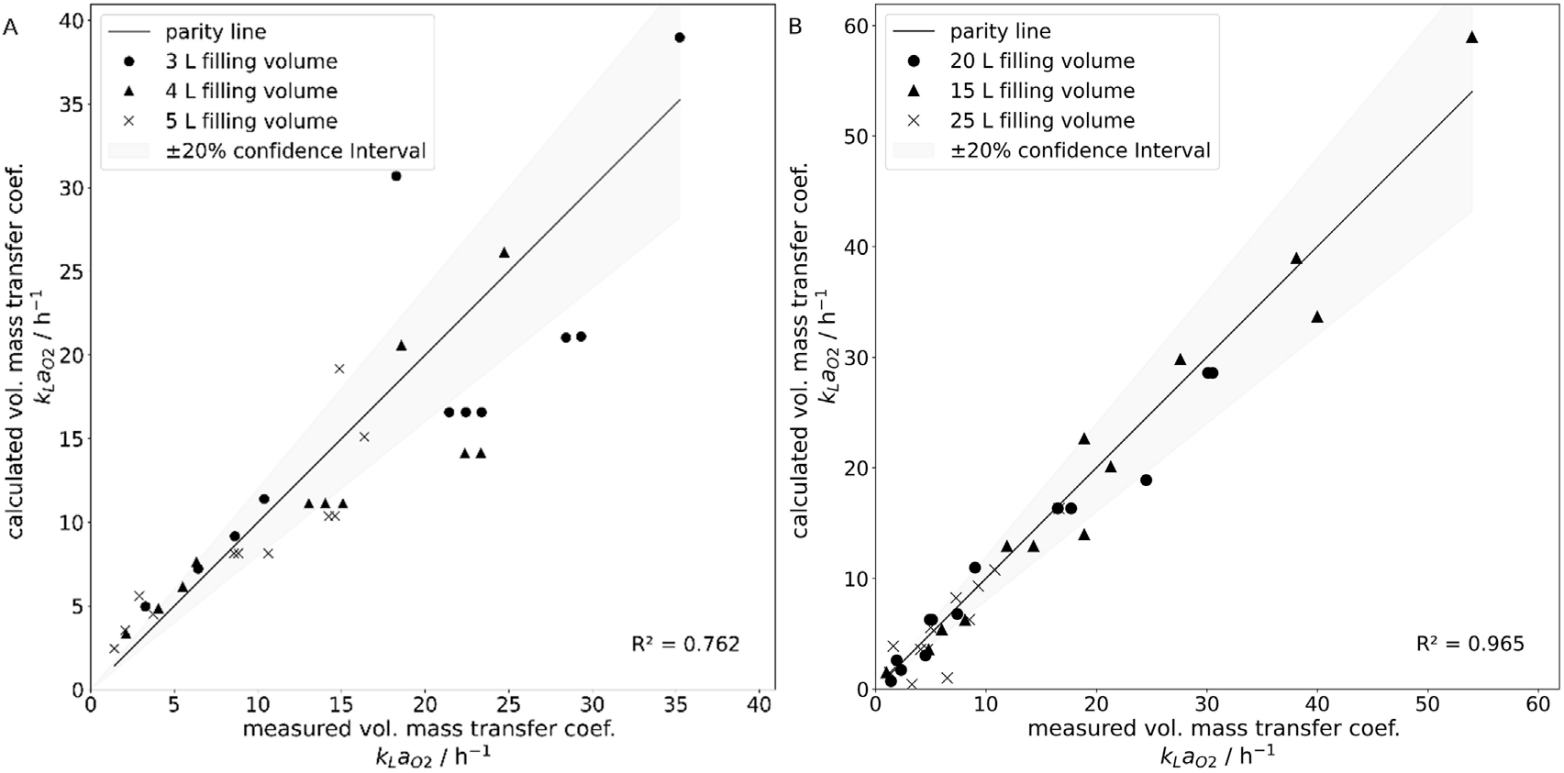
Parity plots of the experimentally determined volumetric mass transfer coefficients shown as data points and the via correlation calculated volumetric mass transfer coefficients, shown as parity line, for the 10 L Wave bioreactor **(A)** for filling volumes 3 L (•), 4 L (▲) and 5 L (x), as well as the 50 L Wave bioreactor **(B)** for filling volumes 15 L (•), 20 L (▲) and 25 L (x). A +/-20% confidence interval was added, known from the experimental error.

The experimental data from the 50 L system aligned more closely with the proposed correlation (R^2^ = 0.959, adjusted R^2^ = 0.956) compared to the 10 L system (R^2^ = 0.736, adjusted R^2^ = 0.710). To illustrate this, the parity plots for the 10 L system (A) and the 50 L system (B) are shown in Figure 9. The confidence interval was derived from the known methodological deviation of ±20% and is indicated accordingly.

The 50 L wave system was identified as a critical step within the process, showing potential for oxygen limitation in future applications due to increasing oxygen demand of new generation cells. This observation suggests that the adjustments of standard operating parameters may be necessary. To address this, the system was used as a basis for optimizing the volumetric mass transfer coefficient using the established correlation equation.

The baseline scenario assumed a rocking rate of *ω* = 24 rpm, a rocking angle of *α* = 7°, and a fill volume of *V* = 20 L, resulting in a calculated mass transfer coefficient of *k*_L_ *a* = 6.57 h^−1^. Two parameter adjustments were evaluated. In the first, the rocking angle was increased from 7° to 9°, while the other parameters remained unchanged. This led to a theoretical increase in vol. mass transfer coefficient to *k*_L_ *a* = 9.70 h^−1^, representing an improvement of approximate 50%. In the second adjustment, both the rocking rate and angle were increased, *ω* = 24 to *ω* = 26 rpm and *α* = 7° to *α* = 9°, respectively, resulting in a theoretical vol. mass transfer coefficient of *k*_L_ *a* = 13.25 h^−1^, effectively doubling the baseline value.

Foam formation emerged as the limiting factor in these adjustments. As both rocking angle and frequency increased, so did foam generation. It was essential to prevent foam from reaching the exhaust filter, as this could lead to blockage, pressure buildup, and potential rupture of the bag. The process was conducted without antifoam agents. During cultivation under elevated operating conditions, foam formation was significantly more pronounced than in the baseline and first adjustment scenarios. Here, no decrease of cell growth was visible due to the potential addition of shear stress. As a result, further increases in operating parameters were not pursued.

Throughout the cultivation process, inline measurements of dissolved oxygen *p*_O2_ were recorded. Additionally, daily sampling was conducted to determine offline dissolved oxygen *p*_O2_, dissolved carbon dioxide *p*_CO2_, and viable cell density *VCD*. Each sampling event was accompanied by a temporary drop in the inline dissolved oxygen, signal, which was consistent with expectations, due to the immediate drop in mass transfer companied by the remaining high demand of oxygen coming from the cells.

Over the three-day cultivation period, a clear difference in oxygen concentration profiles was observed across the different process conditions. In the original culture, a slightly lower dissolved oxygen level was detected at the start, which began to decline rapidly after approximately 1.5 days. By the end of cultivation on day three, the oxygen concentration had dropped to 20% at the time of sampling.

In the run with a single parameter adjustment, the initial dissolved oxygen level was slightly higher than in the benchmark process. Although a decline was also observed over time, the final concentration remained at approximately 40%, twice as high as in the benchmark case. In the process with both parameters adjusted, the initial oxygen level matched that of the single adjustment run. However, the decline was less pronounced, resulting in a final concentration of around 55%, which corresponds to a 175% increase in dissolved oxygen compared to the benchmark.

**FIGURE 10:**
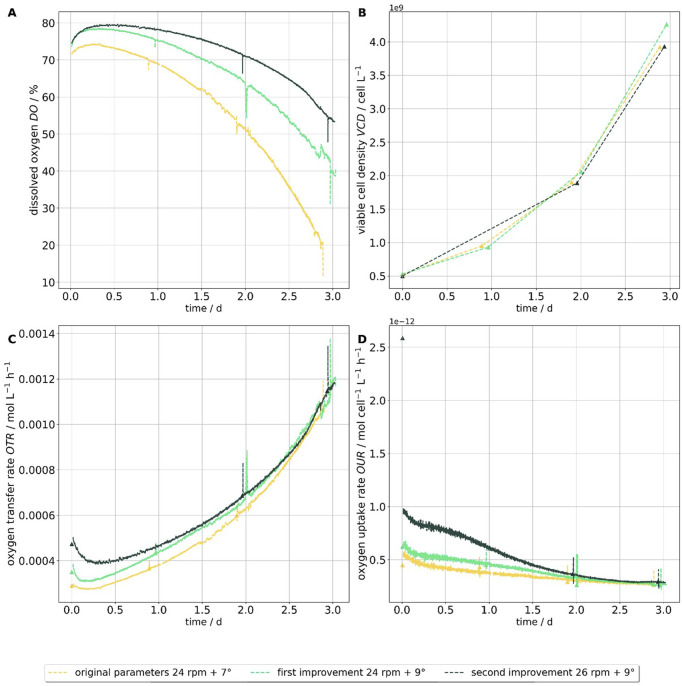
Cultivation data of CHO-K1 cells in a 50 L wave bioreactor with a working volume of 20 L. Three process conditions were tested: a rocking rate of 24 rpm with a rocking angle of 7° (yellow), 24 rpm with 9° (light green), and 26 rpm with 9° (dark green). Subplot **(A)** shows the dissolved oxygen concentration (%), Subplot **(B)** shows the viable cell density *VCD* including measured data points and interpolated values between sampling times. Subplot **(C)** presents the calculated oxygen transfer rate *OTR* based on dissolved oxygen and *VCD*, while subplot **(D)** shows the derived oxygen uptake rate *OUR*.

The viable cell density *VCD* remained largely consistent across all conditions, leading to the conclusion that oxygen transfer rate *OTR* and oxygen uptake rate *OUR* remained consistent as well. The viable cell density in the run with the single parameter adjustment showed a slight increase, though this was not considered significant. All results are shown in Figure 10.

### 3.2 Numerical results

To determine the volumetric mass transfer coefficient, a static flow profile has been developed numerically and after, the volume data for each case were analyzed over one wave cycle, divided into ten time segments. For each time step, the mass transfer coefficient was calculated based on the individual volume fractions and then combined with the specific surface area. The resulting volumetric mass transfer coefficients were averaged over time.

**FIGURE 11:**
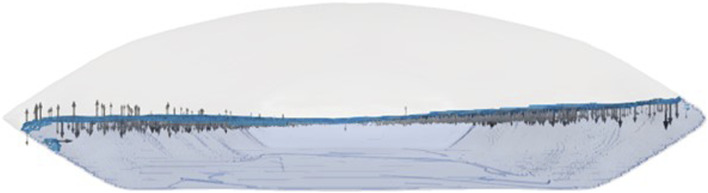
Simulated 50 L wave bioreactor with the liquid phase shown in transparent light blue, the gas–liquid interface highlighted in blue, and the magnitude of the surface normal velocity of the liquid phase indicated by arrows.

Figure 11 shows an exemplary extracted volume. The magnitude of the velocity normal vectors at the surface is indicated by glyphs. Most of these vectors point into the liquid phase, although a smaller number are directed outward. This behavior was attributed to the dynamic motion of the wave, wherein the liquid surface locally shifted in the corresponding direction. Moreover, this phenomenon aligns with the principle of mass conservation, as it is physically implausible for all flow vectors to orient uniformly in the same direction. Overall, the dominant movement was toward the liquid phase.

**FIGURE 12:**
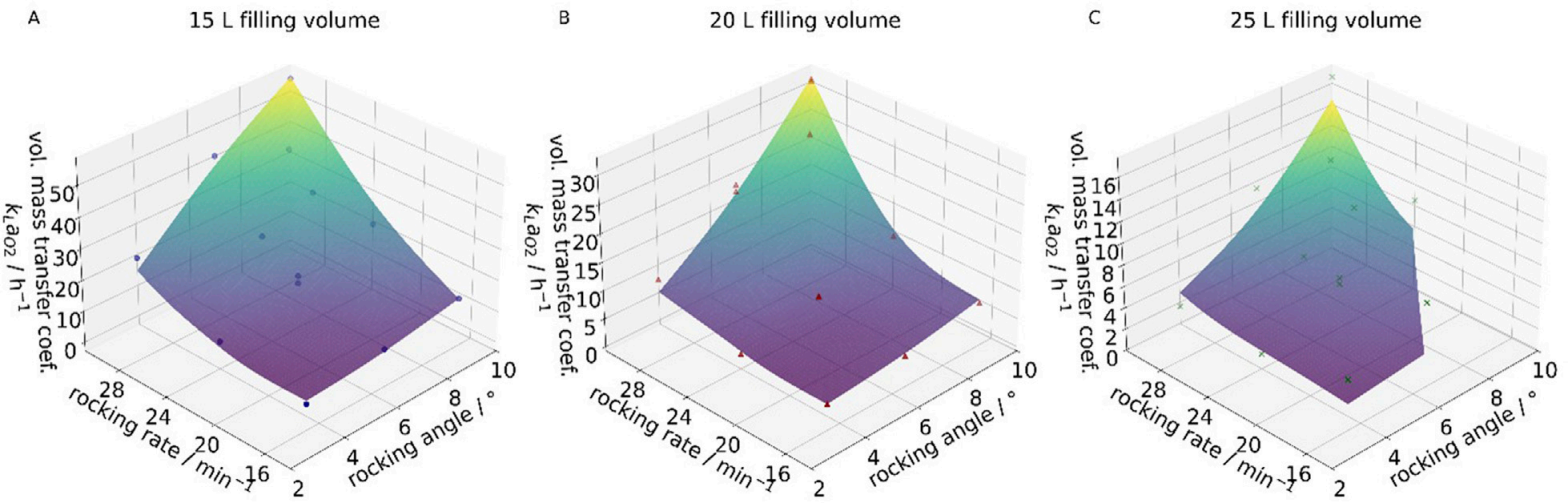
Response surface plots of the correlated data (data points) and the experimental data (surface) for the 50 L wave bioreactors at 15 L filling volume **(A)**, 20 L filling volume **(B)** and 25 L filling volume **(C)**.

The simulations focused primarily on the 50 L system, as it represents a potential limiting factor in future processes, as previously discussed. Simulations were conducted for all cases and compared with experimentally determined data using a response surface plot, segmented by fill volume, as shown in Figure 12.

The model accurately captured the behavior at 20 L and 15 L, while slight deviations were observed at 25 L, particularly at higher rocking angles. This trend closely mirrors the results from the parameter correlation analysis, indicating consistent behavior between the experimental and simulated data.

Simulations were also carried out for the 10 L wave system. However, to reduce computational effort, only boundary conditions and central operating points across all fill volumes were considered. These operating points aligned well with the experimental data. To achieve this agreement, it was necessary to determine a new system-specific fitting parameter, C.

**FIGURE 13:**
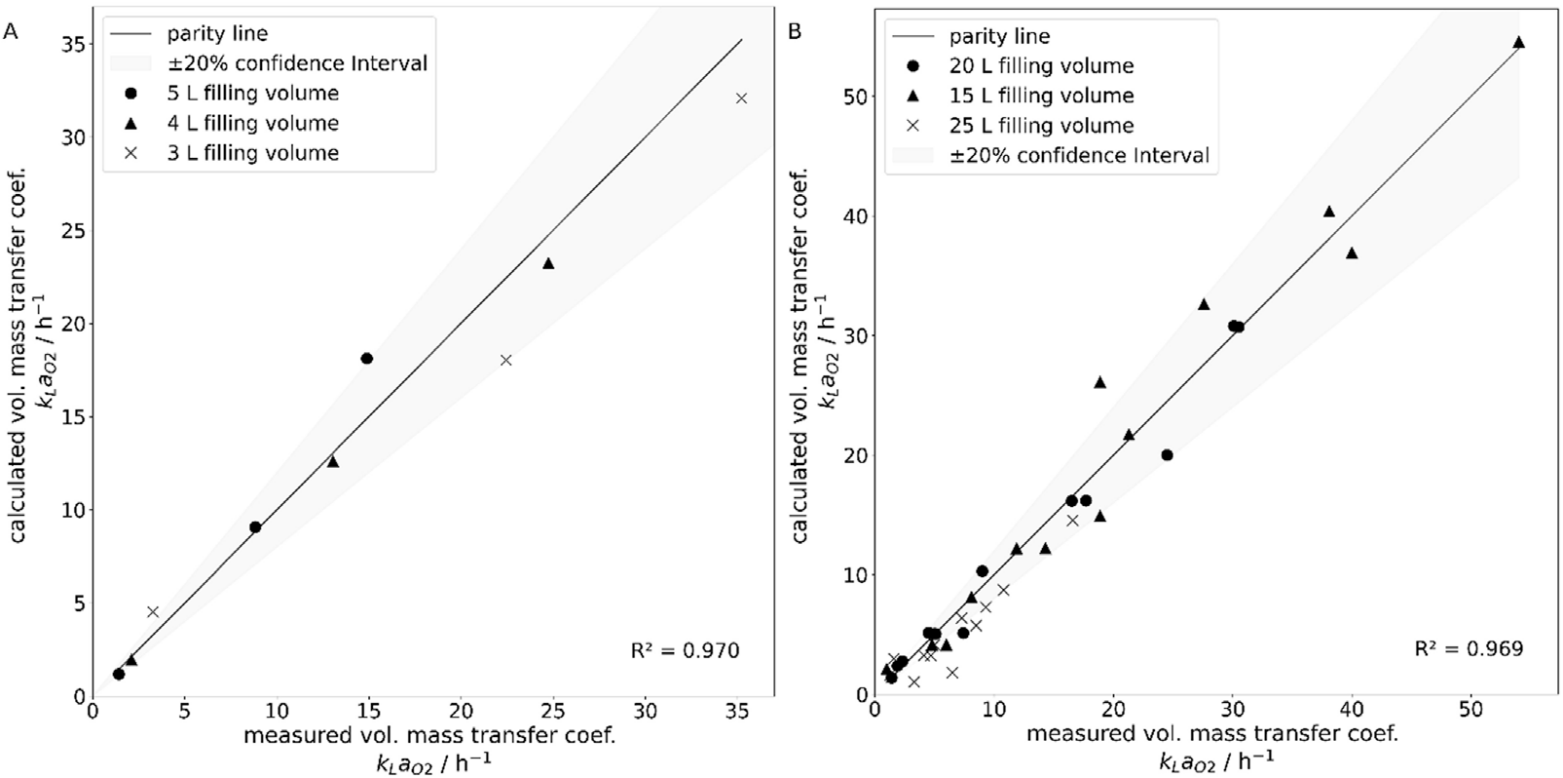
Parity plots of the experimentally determined volumetric mass transfer coefficients shown as parity line and the via simulation calculated volumetric mass transfer coefficients, shown as data points, for the 10 L Wave bioreactor **(A)** for filling volumes 3 L (•), 4 L (▲) and 5 L (x), as well as the 50 L Wave bioreactor **(B)** for filling volumes 15 L (•), 20 L (▲) and 25 L (x). A +/-20% confidence interval was added, known from the experimental error.

Figure 13 presents the parity plots for the 10 L system (A) and the 50 L system (B). Both plots show strong agreement with the experimental results, with only a few data points falling outside the defined confidence interval.

## 4 Discussion

The experimentally determined volumetric mass transfer coefficients for both the 10 L and 50 L systems were generally within the same range as values previously reported under comparable operating conditions (Ghasemi et al., 2018). However, in the referenced study, water was used as the liquid phase, whereas the present work employed PBS supplemented with Pluronic. As a result, the previously reported mass transfer values tended to be higher. This observation aligns with findings by Wierzchowski, who also conducted experiments using wave bioreactors and noted improved mass transfer in pure PBS compared to the values determined here (Wierzchowski et al., 2021). The reduced transfer rates observed in the current study can be attributed to the addition of Pluronic, which is known to negatively affect mass transfer, as reported by Elibol (1999).

The choice of PBS with Pluronic as the liquid phase was based on the results provided, showing that this combination closely resembles the media used for CHO cell cultivation. This enhances the relevance and applicability of the data to actual bioprocesses, especially when compared to studies conducted with water.

In the parameter correlation analysis, both rocking rate and rocking angle had a positive effect on the volumetric mass transfer coefficient, consistent with expectations. In contrast, no influence of the aeration rate was observed, which differs from the findings reported by Pilarek et al. (2018). This discrepancy is likely due to the degassing of the headspace after oxygen removal and before re-aeration. If the headspace is initially filled with nitrogen, the aeration rate significantly affects how quickly the nitrogen is displaced and a stable saturated oxygen concentration *c∗* is established. Since the saturated oxygen concentration *c* ^*∗*^ is typically assumed to be constant, any deviation from this condition over time can impact the calculated mass transfer coefficient. In such cases, the aeration rate would influence how long the saturation oxygen concentration *c* ^*∗*^ remains unstable. However, when the headspace is degassed, the rapid re-inflation with air minimizes differences between the tested aeration rates. Under these conditions, the time required to reach a stable saturated oxygen concentration c^∗^ appears to be short enough that no measurable effect of the aeration rate could be detected. Additionally, fill volume was included as a further process parameter in the correlation equation, as it significantly affects the volume-specific surface area.

The fluid motion observed in the simulation closely matched the behavior seen in real-world experiments. Realistic wave formation was reproduced across various operating conditions. Notably, no resonance effects were detected at low frequencies and angles, consistent with findings from a previous study (Zhan et al., 2019). This phenomenon was also not observed experimentally using the same medium. It should be noted that, under the process conditions investigated in this study, bubble entrainment and wave breaking were not observed and are not expected to contribute significantly to oxygen transfer. The dominant mechanism is surface aeration, enhanced by surface renewal due to the rocking motion. This is consistent with previous reports (Pilarek et al., 2018; Bai et al., 2019a) and our own experimental observations. Our modeling and analysis are therefore focused on these mechanisms. In more rigorous operating regimes, these mechanisms may become relevant, as discussed in (Bai et al., 2019a) but are outside the scope of this study, as they are not relevant for biologicals manufacturing.

Simulations used for fitting must be conducted at the same resolution to ensure consistency, and predictions of mass transfer at other operating points must also be validated at the corresponding resolution. This appears to be a solver-specific effect in M-Star, with similar behavior previously reported for parameters such as shear stress. Further investigation will be necessary to develop improved, M-Star-specific methods for accurately determining the interfacial surface area.

The volumetric mass transfer coefficients calculated from the simulations showed strong agreement with experimental data for both the 10 L and 50 L systems. The analysis was based on an extended form of the Higbie penetration model (Higbie, 1935), originally developed for gas transfer at air bubbles. In its standard form, however, the model does not accurately represent the conditions in wave bioreactors. To better estimate the contact time between air and various points on the gas–liquid interface, the magnitude of the surface-normal velocity was used. Higher normal velocities were interpreted as increased surface renewal due to mass conservation, suggesting that oxygen-depleted fluid is more rapidly replaced, thereby steepening the concentration gradient and enhancing mass transfer. The use of a characteristic velocity to estimate the volumetric mass transfer coefficient was previously proposed by Friedl, who demonstrated the influence of contact velocity on mass transfer of a wave induces liquid within a wind tunnel (Friedl, 2013).

## 5 Conclusion

This study presents a comprehensive experimental and computational investigation of oxygen transfer in wave bioreactors, focusing on both 10 L and 50 L single-use systems. An improved experimental method was developed to determine the volumetric mass transfer coefficient addressing limitations of previous approaches by ensuring ideal gas phase mixing and including filling volume as a key parameter. The results show that rocking rate, rocking angle, and filling volume all influence oxygen transfer. Notably, filling volume was included as a process parameter in this correlation, which sets this work apart from previous studies that did not account for its effect on the interfacial area (Pilarek et al., 2018).

Through a full factorial design of experiments and correlation analysis, robust predictive models for the volumetric mass transfer coefficient in both wave bioreactor scales were established. These models were validated in biological cultivations, where optimized process parameters led to significantly increased dissolved oxygen levels without compromising cell growth or viability. Notably, foam formation was identified as a practical limitation when increasing rocking intensity, highlighting the need to balance oxygen mass transfer enhancement with operational constraints and putting the finding into the context of real-world applicability.

A Lattice-Boltzmann Method based CFD approach was used to simulate fluid dynamics and mass transfer. The volumetric mass transfer calculations based on the simulations, which included ideas from the surface renewal theory (Kermani et al., 2011), penetration theory (Higbie, 1935) and findings by Friedl (2013), matched experimental data and provided insight into oxygen transfer mechanisms at the gas–liquid interface, using the surface-normal velocity as an important factor.

These results advance the understanding of oxygen transfer in wave bioreactors, offer validated models for process optimization, and demonstrate the value of LBM-based CFD in bioprocess engineering. The insights support improved design and scale-up of wave bioreactor processes in biopharmaceutical manufacturing.

## Data Availability

The raw data supporting the conclusions of this article will be made available by the authors, without undue reservation.

## Glossary

### Abbreviations

CFD: Computational Fluid Dynamics
CHO: Chinese Hamster Overy
DOE: Design of experiments
LBM: Lattice-Boltzmann-Method
DO: dissolved oxygen
PBS: phosphate buffer solution
RANS: Reynolds-Averaged Navier–Stokes
VOF: Volume of Fluid

### Symbols

α: rocking angle/°
**α**_**1**_: fitting parameter/-
**α**_**L**_: volume fraction range/-
**a**_**i**_: area of lattice cell/m^2^
**β**_**1**_: fitting parameter/-
**β**_**L**_: mass transfer resistance/s·m^−1^
c: measured oxygen concentration/mol·L^−1^
c*: saturated oxygen concentration/mol·L^−1^
**C**_**1**_: fitting parameter/-
c: fitting parameter/-
Co: Courant Number/-
**d**_**s**_: shaker diameter/m
**d**_**v**_: vessel diameter/m
**D**_**L**_: diffusion coefficient/m^2^·s^−1^
DO: dissolved oxygen/%
ε: eddy dissipation rate
g: gravity/m·s^−2^
h: filling height/m
i: lattice cell/-
γ: fitting parameter/-
**k**_**L**_ **a**: volumetric mass transfer coefficient/h^−1^
**k**_**L**_: mass transfer coefficient/h^−1^
L: length of wave bioreactor in x direction/m
**L**_**x**_: resolution/m^−1^
n: shaking frequency/s^-1^
OTR: oxygen transfer rate/mol·L^−1^·h^−1^
OUR: specific oxygen uptake rate/mol·cell^−1^·L^−1^·h^-−1^
P: power input/W
q: air ration rate/L·h^−1^
r: radius/m
t: time/s
V: filling volume/L
VCD: viable cell density/cell·L^−1^
v: velocity of wave bioreactor movement/m·s^−1^
**v**_**n**_: magnitude of normal velocity/m·s^−1^
z: velocity of wave bioreactor movement in z direction/m·s^−1^
ω: rocking rate/s^−1^
