# Supplementary material for: Modeling and validating of oxygen transport in wave bioreactors: optimized experimental mass transfer method and novel Lattice-Boltzmann CFD approach
Source: Front Bioeng Biotechnol. 2026 Jan 21;13:1688774. doi: 10.3389/fbioe.2025.1688774 (PMC12868168; doi:10.3389/fbioe.2025.1688774)
Supplement: Supplementary file 1 [file DataSheet2.pdf]

Tested Parameters

10 L Wave bioreactor

case	rocking rate / min^{-1}	rocking angle / °	filling volume / L
1	24	7	5
2	24	7	4.7
3	24	9	4.7
4	26	9	4.7
5	17	4	5
6	31	10	5
7	17	4	4
8	24	7	4
9	31	4	4
10	31	10	4
11	17	4	3
12	17	10	3
13	24	7	3
14	31	10	3
15	31	4	3
16	31	4	5
17	17	10	5
18	17	10	4

50L Wave bioreactor

case	rocking rate / min^{-1}	rocking angle / °	filling volume / L
1	24	7	20
2	17	4	20
3	17	7	20
4	17	10	20
5	24	4	20
6	24	10	20
7	31	4	20
8	31	7	20
9	31	10	20
10	24	7	20
11	24	7	20
12	31	7	20
13	31	10	20
14	29	9	20
15	17	4	15
16	24	7	15
17	29	9	15
18	27	7	15
19	27	9	15
20	24	7	25
21	29	9	25
22	27	7	25
23	27	9	25
24	24	7	25
25	17	4	25
26	17	7	25
27	17	10	25
28	24	4	25
29	24	10	25
30	31	4	25
31	31	7	25
32	31	10	25
33	24	7	15
34	17	4	15
35	17	7	15
36	17	10	15
37	24	4	15
38	24	10	15
39	31	4	15
40	31	7	15
41	31	10	15
