# Supplementary material for: Modeling and validating of oxygen transport in wave bioreactors: optimized experimental mass transfer method and novel Lattice-Boltzmann CFD approach
Source: Front Bioeng Biotechnol. 2026 Jan 21;13:1688774. doi: 10.3389/fbioe.2025.1688774 (PMC12868168; doi:10.3389/fbioe.2025.1688774)
Supplement: Supplementary file 3 [file DataSheet6.pdf]

```
#this is the script used for the data analysis of the 50 L wave.

import numpy as np
from scipy.optimize import minimize
from scipy.optimize import differential_evolution
import time
import os
import matplotlib.pyplot as plt

def objective(params, normal_vel_data_all,diffusionCoeff,nu, EDR_data_all,
gradient_data_all, kla_data_all):
    C = params [0]

    result            =            (((normal_vel_data_all/C)            *
(((diffusionCoeff*((EDR_data_all/nu)**0.5))**0.5)))*3600*gradient_data_al
l - kla_data_all)/kla_data_all

    mean_result = np.mean(result)


    return (mean_result)**2


diffusionCoeff = 2.1 * 10 ** (-9)  # Diffusion coefficient in m²/s
nu = 10 ** (-6)

case_name = [1, 2, 3, 4, 5, 6, 7, 8, 9, 10, 11, 12, 13, 14, 15, 16, 17,
18, 19, 20, 21, 22, 23, 24, 25, 26, 28, 29, 30, 31, 32, 33, 34, 35, 36,
37, 38, 39, 40, 41]
cases_to_evaluate = [i for i in range(1, 42) if i != 27]



if os.name == 'nt':  # Windows
    volume_file_paths = [
        rf"path to raw data"
        for i in case_name
    ]
else:  # Linux or other
    volume_file_paths = [
        rf"path to raw data"
        for i in case_name
    ]

"""kLa_exp = [5.0, 1.4, 2.3, 4.5, 1.9, 9.0, 7.4, 17.7, 30.1, 5.1, 4.9,
16.5, 30.5, 24.5,
    1.0, 11.9, 38.1, 21.3, 27.6, 4.1, 10.8, 5.0, 7.3, 4.7, 3.3, 6.5, 1.3,
8.5, 1.6, 9.3, 16.6, 14.3, 1.0, 4.8, 8.1, 6.0,
    18.9, 18.9, 40.0, 54.0, 54.0, 54.0, 9.0]"""
kLa_exp = [5.0, 1.4, 2.3, 4.5, 1.9, 9.0, 7.4, 17.7, 30.1, 5.1, 4.9, 16.5,
30.5, 24.5,
    1.0, 11.9, 38.1, 21.3, 27.6, 4.1, 10.8, 5.0, 7.3, 4.7, 3.3, 6.5, 1.3,
8.5, 1.6, 9.3, 16.6, 14.3, 1.0, 4.8, 8.1, 6.0,
    18.9, 18.9, 40.0, 54.0]


def    fit_parameter(objective,    diffusionCoeff,    nu,    case_name,
cases_to_evaluate, volume_file_paths, kLa_exp):
```

```
    gradient_data_all = np.array([])
    normal_vel_data_all = np.array([])
    EDR_data_all = np.array([])
    volume_data_all = np.array([])
    kla_data_all = np.array([])

    for idx, file_path in enumerate(volume_file_paths):
        if case_name[idx] in cases_to_evaluate:
            if 1 <= case_name[idx] <= 14:
                volume = 20
            elif 15 <= case_name[idx] <= 19:
                volume = 15
            elif 20 <= case_name[idx] <= 32:
                volume = 25
            elif 33 <= case_name[idx] <= 42:
                volume = 15
            if kLa_exp[idx] == 0:
                continue
            Z = kLa_exp[idx] * volume / 1000
            try:
                start_time = time.time()
                print(f"Loading file {case_name[idx]}")
                data = np.load(file_path)
                end_time = time.time()
                elapsed_time = end_time - start_time
                data_size = sum(data[key].nbytes for key in data)
                print(f"File {case_name[idx]} loaded successfully in
{elapsed_time:.2f} seconds")
                print(f"Data size: {data_size / (1024 * 1024):.2f} MB")
            except Exception as e:
                print(f"Error loading file {file_path}: {e}")
                continue

            gradient_data_all = np.append(gradient_data_all,
data['gradient_data_all'])
            normal_vel_data_all = np.append(normal_vel_data_all,
data['normal_vel_data_all'])
            EDR_data_all = np.append(EDR_data_all, data['EDR_data_all'])
            temp = np.ones(len(data['normal_vel_data_all'])) * volume
            volume_data_all = np.append(volume_data_all, temp)
            temp = np.ones(len(data['normal_vel_data_all'])) * Z
            kla_data_all = np.append(kla_data_all, temp)


    initial_guess = [0.4]

    # Optimierung durchführen
    start_optimization_time = time.time()

    #initial_guesses = [0.4, 1.0, 10.0]

    """for initial_guess in initial_guesses:
        result = minimize(objective, [initial_guess], method='Nelder-
Mead', args=(normal_vel_data_all, diffusionCoeff, nu, EDR_data_all,
gradient_data_all, kla_data_all))
        print(f"Initial Guess: {initial_guess}, Optimized C: {result.x},
Objective Value: {result.fun}")"""
```

```
    result = minimize(objective, initial_guess, method='Nelder-Mead', args=(normal_vel_data_all, diffusionCoeff, nu, EDR_data_all, gradient_data_all, kla_data_all))
    #bounds = [(80, 90)]  # Adjust bounds as needed
    #result = differential_evolution(objective, bounds, args=(normal_vel_data_all, diffusionCoeff, nu, EDR_data_all, gradient_data_all, kla_data_all))
    end_optimization_time = time.time()
    optimization_elapsed_time = end_optimization_time - start_optimization_time
    print(f"Optimization completed in {optimization_elapsed_time:.2f} seconds")

    # Optimierte Werte für C
    C_opt = result.x

    return C_opt

C_opt = fit_parameter (objective, diffusionCoeff, nu, case_name, cases_to_evaluate, volume_file_paths, kLa_exp)


print("Optimierte Werte:")
print("C:", C_opt)

num_kla_all = np.zeros(len(case_name))
for idx, file_path in enumerate(volume_file_paths):
    gradient_data_all = np.array([])
    normal_vel_data_all = np.array([])
    EDR_data_all = np.array([])
    volume_data_all = np.array([])
    kla_data_all = np.array([])
    if case_name[idx] in cases_to_evaluate:
        if 1 <= case_name[idx] <= 14:
            volume = 20
        elif 15 <= case_name[idx] <= 19:
            volume = 15
        elif 20 <= case_name[idx] <= 32:
            volume = 25
        elif 33 <= case_name[idx] <= 42:
            volume = 15
        if kLa_exp[idx] == 0:
            continue
        Z = kLa_exp[idx] * volume / 1000
        try:
            start_time = time.time()
            print(f"Loading file {case_name[idx]}")
            data = np.load(file_path)
            end_time = time.time()
            elapsed_time = end_time - start_time
            data_size = sum(data[key].nbytes for key in data)
            print(f"File {case_name[idx]} loaded successfully in {elapsed_time:.2f} seconds")
            print(f"Data size: {data_size / (1024 * 1024):.2f} MB")
        except Exception as e:
            print(f"Error loading file {file_path}: {e}")
            continue
```

```
        gradient_data_all = np.append(gradient_data_all,
data['gradient_data_all'])
        normal_vel_data_all = np.append(normal_vel_data_all,
data['normal_vel_data_all'])
        EDR_data_all = np.append(EDR_data_all, data['EDR_data_all'])
        temp = np.ones(len(data['normal_vel_data_all'])) * volume
        volume_data_all = np.append(volume_data_all, temp)
        temp = np.ones(len(data['normal_vel_data_all'])) * Z
        kla_data_all = np.append(kla_data_all, temp)

        result = ((normal_vel_data_all/C_opt) *
(((diffusionCoeff*((EDR_data_all/nu)**0.5))**0.5)))*3600*gradient_data_al
l
        num_kla_all[idx] = np.mean(result) / volume * 1000
        # Calculate R² for each case
        mean_gradient = np.mean(gradient_data_all)
        mean_result = np.mean(result / 3600 / gradient_data_all)
        print(f"Mean gradient_data_all: {mean_gradient}")
        print(f"Mean of (result / 3600 / gradient_data_all):
{mean_result}")
        print(f"Case {case_name[idx]}: {num_kla_all[idx]} vs.
{kLa_exp[idx]}")
        #r_squared = 1 - (np.sum((kLa_exp[idx] - num_kla_all[idx]) ** 2) /
np.sum((kLa_exp[idx] - np.mean(kLa_exp)) ** 2))
        #print(f"R² for case {case_name[idx]}: {r_squared}")

    # Calculate the mean R² for all cases
mean_r_squared = 1 - (np.sum((np.array(kLa_exp) - num_kla_all) ** 2) /
np.sum((np.array(kLa_exp) - np.mean(kLa_exp)) ** 2))
# Print all calculated kLa values
print("All calculated kLa values:")
print(num_kla_all.tolist())

# If you want to see them with case numbers for better reference
kla_with_cases = {case_name[i]: num_kla_all[i] for i in
range(len(case_name))}
print("kLa values with case numbers:")
print(kla_with_cases)
print(f"Mean R² for all cases: {mean_r_squared}")

parity_plot = plt.figure(figsize=(12, 12))  # Set square figure size


line_color = '#333333'  # Dark grey for parity line
marker_color = '#000000'  # Black for markers
confidence_interval_color = '#E5E3DE'  # Light grey for confidence interval
Fontsize = 28  # Increased font size for labels
Fontsize2 = 24  # Increased font size for legend
AxisFontsize = 28  # Increased font size for axes

plt.plot(kLa_exp, kLa_exp, '-', label='parity line', color=line_color)
plt.plot(kLa_exp, num_kla_all, 'o', label='numerical vol. mass transfer
coef.', color=marker_color)

# Adding confidence interval
# Sort data to properly fill the confidence interval area
sort_idx = np.argsort(kLa_exp)
kLa_exp_sorted = np.array(kLa_exp)[sort_idx]
```

```
lower_bound = kLa_exp_sorted * 0.8
upper_bound = kLa_exp_sorted * 1.2
plt.fill_between(kLa_exp_sorted,        lower_bound,        upper_bound,
color=confidence_interval_color,   alpha=0.2,   label=f'±20%   confidence
Interval')

plt.ylabel('numerical  vol.  mass  transfer  coef.  \n$k_{L}a_{O2}$  /  h$^{-
1}$', fontsize=Fontsize)
plt.xlabel('measured vol. mass transfer coef. \n$k_{L}a_{O2}$ / h$^{-1}$',
fontsize=Fontsize)
plt.xticks(fontsize=AxisFontsize)
plt.yticks(fontsize=AxisFontsize)

# Make plot square with equal aspect ratio
plt.gca().set_aspect('equal')

# Set the same limits for both axes to ensure square appearance
max_val = max(max(kLa_exp), max(num_kla_all)) * 1.05
plt.xlim(0, max_val)
plt.ylim(0, max_val)

plt.legend(fontsize=Fontsize2, loc='upper left')
plt.tight_layout()  # Adjust layout to make room for labels
#parity_plot.patch.set_facecolor('#F6F5F3')  # Set background color to grey
color
#parity_plot.patch.set_facecolor('#FFFFFF')  #  Set  background  color  to
white color

plt.savefig(r'path  to  save  figure',  dpi=300,  bbox_inches='tight')     #
Higher DPI for better quality
plt.show()
```
