# Supplementary material for: Modeling and validating of oxygen transport in wave bioreactors: optimized experimental mass transfer method and novel Lattice-Boltzmann CFD approach
Source: Front Bioeng Biotechnol. 2026 Jan 21;13:1688774. doi: 10.3389/fbioe.2025.1688774 (PMC12868168; doi:10.3389/fbioe.2025.1688774)
Supplement: Supplementary file 6 [file DataSheet3.pdf]

Additional Data for Simulation setup and fluid properties

fluid properties are measured properties based on the media composition provided.

Properties		
density	1000	kg/L
diffusion coefficient	2.1*10^-9	m^2/s
surface tension	0.072	N/m
kinematic Viscosity	1*10^-6	m^2/s
contact angle assumptions	90°	
Boundry Type	Grid Aligned	
Fluid Interaction	on	
