# Supplementary material for: Modeling and validating of oxygen transport in wave bioreactors: optimized experimental mass transfer method and novel Lattice-Boltzmann CFD approach
Source: Front Bioeng Biotechnol. 2026 Jan 21;13:1688774. doi: 10.3389/fbioe.2025.1688774 (PMC12868168; doi:10.3389/fbioe.2025.1688774)
Supplement: Supplementary file 7 [file DataSheet1.pdf]

Composition of PBS (Phosphate-Buffered Saline):

8 g/L Sodium Chloride

1.434 g/L Disodium Hydrogen Phosphate

0.2 g/L Potassium Chloride

0.2 g/L Potassium Dihydrogen Phosphate

1 g/L Pluronic
